# Research on the influence of college students’ participation in sports activities on their sense of inferiority based on self-esteem and general self-efficacy

**DOI:** 10.3389/fpsyg.2022.994209

**Published:** 2022-11-10

**Authors:** Chu Liu

**Affiliations:** College of Physical Education, Northeast Normal University, Changchun, Jilin, China

**Keywords:** sports activities, sense of inferiority, self-esteem, general self-efficacy, college students

## Abstract

College students need to face various problems and conflicts, and are prone to many negative emotions, such as depression, low self-esteem, social anxiety, low sense of belonging, lack of self-identity, and so on. The generation of these bad emotions will bring unexpected negative effects to college students. Taking Chinese college students as the research object, this study analyzes the influence of sports participation on inferiority. Furthermore, we explored whether self-esteem and general self-efficacy mediated the relationship between physical activity and inferiority. In this study, 115 students were selected to conduct the experiment for 12 weeks. After reliability testing, the collected data were analyzed by multivariate analysis of variance to verify the research model. The results show that sports has a significant positive correlation with the reduction of college students’ inferiority complex. What is important in this model is that self-esteem and general self-efficacy are enhanced during physical activity and decreased during inferiority complex. In addition, this study created three sports situations: competition group, entertainment group and control group. The comparison results show that competitive sports situation is better than leisure sports situation in terms of the influence on college students’ inferiority complex. Through the theoretical and empirical research on college students’ inferiority complex, it is concluded that sports is an effective means to reduce college students’ inferiority complex.

## Introduction

Young people are healthy in body and mind, strong in body, strong in will, and full of vitality. They are a reflection of the great vitality of a nation, a symbol of social progress, and civilization, and a major event concerning the future of the country and the nation (Walls et al., 2012). College students are prone to various mental health problems due to increasing learning pressure, employment pressure, interpersonal communication confusion, and the sharp contrast of ideological changes caused by the weakening or loss of advantages in the same group after entering the university (Lola and Tzetzis 2020). Since the rise of exercise psychology in 1960s and 1970s, the study of sports exercise and mental health has become an important field of sports psychology (Amp and Morris, 2011; Tülay and Ejder, 2018). Zeng and Li (2022) pointed out that the main purpose of exercise psychology research is to promote physical and mental health and form a good physical and mental state. The research focuses on how to maintain and improve physical and mental health and form a good physical and mental state through various sports exercises, to help athletes and exercisers benefit mentally, emotionally, and cognitively from sport activity. Searching the literature, there is no research on the influence of physical exercise and inferiority feeling at present. Because long-term sense of inferiority is one of the important reasons leading to various psychological problems of college students, sense of inferiority is also an important factor affecting the growth of college students (Jianbo and Aihua, 2019). Estrella et al. (2013) pointed out that in recent years, researches on college students’ inferiority complex have attracted great attention from scholars. A large number of studies have shown that sports activities can affect people’s cognition, self-esteem, self-efficacy, coping style, attribution style, interpersonal relationship, personality traits, and so on, and there is a close relationship between sense of inferiority and the above factors, so it is very possible that sports activities can have a certain impact on sense of inferiority. This study has important theoretical and practical significance. From the theoretical sense, this research on college students’ sense of inferiority, the general differences, the relationship between college students’ sense of inferiority and sports activities, all these studies will help to improve the recognition of this psychological phenomenon on college students’ sense of inferiority levels. From the practical significance, the research on college students’ sense of inferiority and its correlation with sports activities can better understand the relationship between sports activities and college students’ mental health, and provide exercise basis and practical guidance for sports activities to maintain college students’ mental health. However, there are not many specific studies on the influence of sports activities on college students’ inferiority. To sum up, this study aims to study the relationship between college students’ feelings of inferiority and physical activities. From the theoretical point of view, it is hoped that these studies can improve college students’ understanding of inferiority complex. In a practical sense, the study of college students’ inferiority complex and its correlation with sports activities can provide exercise basis and practical guidance for sports activities to maintain college students’ mental health.

## Related research and theoretical framework

### Related work

#### Self-esteem and self-efficacy

Compared with adults, college students are at higher risk of poor mental health. A series of factors such as family, school, study, and social interaction may induce college students’ mental health problems (Liu et al., 2022). Liu et al. (2022) pointed out that anxiety level was negatively correlated with self-esteem in campus life, and increased with the increase of school years. However, there is no consensus among scholars in the studies on depression, anxiety, and stress of college students. Liu et al. (2019) used scale analysis and pointed out that the highest scores of depression, anxiety and stress appeared in the first or second year on average. In terms of gender, women have a higher increased risk than men (Gao et al., 2022). From a more macroscopic perspective, Liu et al. (2022) reviewed the existing literature on non-drug intervention of college students’ mental health. In addition, the research of self-esteem and self-efficacy is also the object of scholars’ attention, self-esteem and self-efficacy affect people’s efforts to achieve goals. Luo et al. (2022) verified that students’ self-esteem can predict their subsequent academic self-efficacy. Zhang et al. (2022) pointed out that self-esteem and general self-efficacy are mutually reinforcing among students in elite universities. In particular, there is a stronger correlation between self-esteem and self-efficacy in female college students (Tus, 2020).

#### Sports

There is no doubt that proper physical exercise is good for people’s physical and mental health. Many scholars have confirmed the positive effect of sports on college students’ personal development. Muñoz-Bullón et al. (2017) compared the academic performance of sports participants with that of non-participants and found that participation in formal sports had a positive impact on students’ higher grades. In terms of the psychological impact of sports encounters on students, de Souza et al. (2021) compared sports students with medical students and pointed out that medical students had higher anxiety values than sports students during the COVID-19 epidemic. This may be because physical activity reduces trait anxiety (Felez-Nobrega et al., 2020). Similarly, Uddin et al. (2020), in a one-year prospective study of university students in Dhaka, Bangladesh, pointed out that physical inactivity was associated with high psychological distress among university youth in Bangladesh.

The current research is more accurate to the relationship between sports and the mental health of college students. Inferiority complex is a part of college students’ mental health. Few scholars have explored the relationship between inferiority complex and college students’ sports. However, it is worth pondering whether sports can reduce the inferiority of college students? What role does self-esteem and general self-efficacy play? Based on this, this study analyzes sports, self-esteem, self-efficacy, and inferiority complex. We hope to help reduce the inferiority of college students.

### Theoretical framework

#### Sports activities

It can be seen from the research results in the literature that there are different views on the definition of sports activities due to different perspectives and research purposes. Although the expressions are different, the basic connotations are similar (Ruiz et al., 2019).

According to Scheper et al. (2013), sport activity is a kind of physical exercise that takes physical exercise and exercise load as the means, fitness, recreation, health care, and rehabilitation, mental intelligence exercise as the content, and for the purpose of enhancing physical fitness and improving physical and mental health, improves and maintains the body’s ability. Ji and Zheng (2021) pointed out that sports activities include various forms of activities related to cardiopulmonary function, muscle strength and endurance, flexibility and body composition, and so on. It usually refers to those planned, regular and repetitive sports activities aimed at enhancing physical fitness. In order to meet the needs of research, according to the actual situation of college students in physical education, college physical education teaching, extracurricular sports activities and exercise are included in the category of college students’ sports activities. Sports activities scene mainly refers to the synthesis of external conditions in the inner world of exercisers caused by the exercise place.

#### Sense of inferiority

Sense of inferiority is a very familiar concept in people’s social life as well as one of the more frequently used concepts in psychology. The different definitions of sense of inferiority reflect the different emphases and research methods used by psychologists on inferiority feelings (Tian and Shi, 2022). As for the concept of sense of inferiority, many scholars have carried out in-depth exploration, but often from their own research Angle, so there are different understandings, which leads to the ambiguity of sense of inferiority. Chen et al. (2020) believed that sense of inferiority is a psychological component with a complex structure. It is not only an individual’s negative evaluation of himself, but also an emotional experience of rejecting himself accompanied by and based on negative evaluation. Yang et al. (2014) defined sense of inferiority as a psychological phenomenon caused by low self-evaluation and corresponding negative psychological experience. This study summarizes the definition of sense of inferiority as inferiority is not only a negative evaluation of the self, but also an emotional experience of rejecting the self that is accompanied by and based on negative evaluation. That is, low self-evaluation, and the resulting negative emotional state.

#### Self-esteem

Self-esteem is one of the core components of individual self-system, which is not only directly related to individual mental health, but also has a wide impact on individual cognition, motivation, emotion, and behavior. At present, there is no unified conclusion on the definition of self-esteem. Researchers have different perspectives, different research purposes, and different levels of self-esteem involved, so they have different understandings of self-esteem. Here, several representative definitions at home and abroad are briefly introduced. Joseph et al. (2014) believed that self-esteem is a reflection of the relationship between social evaluation and personal self-esteem needs. It is pointed out that self-esteem is based on self-image and understanding of one’s social value, and it is an evaluation of one’s degree of respect or importance. Melnyk et al. (2014) believed that self-esteem is the sense of self-worth, namely, one. It is a positive self-emotional experience that an individual recognizes and evaluates himself as an object to the social body, including the group, others and himself as an individual in social life. Although these definitions have different emphases, they all show that self-esteem is a kind of value judgment involving individual self-cognition, and a positive emotional experience of individual’s own value, importance, significance, and success.

#### General self-efficacy

Self-efficacy was proposed by Albert Bandura, the founder of American social learning theory (Benlian and Yang, 2013). It can describe a person’s ability to perform a certain behavior in a particular situation and achieve the desired result. To a large extent, self-efficacy refers to an individual’s sense of self-related abilities. If a person can believe that he can handle various things well, he will be more active and more active in life, work and study. Zhaohui (2020) pointed out that general self-efficacy has an impact on people’s social behaviors and plays a certain role in controlling people’s behavior patterns in the environment. It can be said that self-efficacy is directly related to individual behavior patterns and psychological emotions. At present, general self-efficacy has been more and more applied to the mental health of different people, and has been studied in many fields such as interpersonal communication, higher education, and organization. The higher the level of general self-efficacy, the greater the achievement of an individual’s behavior. Therefore, people with high general self-efficacy view and deal with various pressures in life better through higher self-confidence or better self-feeling.

#### Implementation path

Many research results show that under the same condition of sports intensity, exercise time, sports frequency and sports type, competitive exercise situation, and self-choice sports content situation are more conducive to the reduction of anxiety level of students (Su et al., 2015). Some researchers put forward that in order to enlarge the psychological effect of sports activities, it is necessary to avoid the competition between people in exercise. Therefore, in order to achieve greater psychological effect of sports, it is necessary to study the influence of sports situation on sports effect. In recent years, many scholars in the field of sports and exercise psychology have carried out experimental studies on the influence of sports on many aspects of mental health (such as anxiety, depression, personality, and so on), but there is no literature on the effect of sports on reducing sense of inferiority (Conroy et al., 2013).

**Figure 1:**
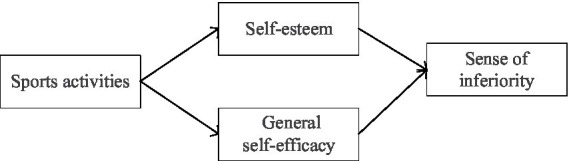
The framework model.

The above theoretical framework explains the definition and influencing factors of the key concepts in this paper. Furthermore, we constructed a framework model of physical activity, inferiority complex, self-esteem, and general self-efficacy. As shown in Figure 1.

As the object of this study for college students, in the current higher education sports and health curriculum reform, very emphasis on sports education teaching methods to arouse students’ interest in sports, insist on sports habit, cultivate lifelong sports consciousness, the formation of courageous tenacity and perseverance will quality, promote students in the physical, psychological and social adaptation ability and so on healthy and harmonious development. This study not only helps to understand the relationship between different sports situations and the psychological effects of sports, but also help to provide experimental support for the change of learning methods proposed in the PE curriculum reform of higher education. At the same time, this study attempts to verify the mediating role of self-esteem and general self-efficacy variables in the influence of sports on college students’ sense of inferiority, that is to further verify the psychological mechanism model of sports on reducing college students’ sense of inferiority.

## Research object and method

### Research object

A questionnaire survey was conducted on the students of Northeast Normal University, and the scores of students’ inferiority feeling were ranked from high to low. Among the students whose inferiority feeling score was higher than the average score, 110 students were selected to participate in this experiment voluntarily, including 62 males and 48 females. We must emphasize that explaining to the respondents the topic of filling in the questionnaire and the meaning contained in each question is a key step that cannot be ignored. We explain ed. to each respondent the meaning of each question in the questionnaire. The basic information of the research object is shown in Table 1.

**Table 1:** Basic information of the research object.

General information		Number of samples	Proportion (%)
Gender	Male	62	56.36
	Female	48	43.64
Grade	Freshman	30	27.27
	Sophomore	26	23.64
	Junior	18	16.36
	Senior	36	32.73
Major	Liberal arts	41	37.27
	Science and engineering	66	60.00
	Others	3	2.73

### Research method

#### Grouping method

The students’ sense of inferiority level, self-esteem, general self-efficacy and so on were pre-tested, and the students who scored higher than the average score were marked. According to the gender characteristics and inferiority score of the objects. The subjects were randomly divided into three groups. The three groups were named competition group, entertainment group and control group. Different groups adopted different practice styles. The grouping table is shown in Table 2.

**Table 2:** Grouping table.

Groups	Number of samples	Sports content
Competition group	37	Combine technique with competition practice
Entertainment group	37	Combine techniques with recreational exercises
Control group	36	No specific requirements
P	> 0.05	–

In the experiment, participants in the competition group and the entertainment group of each item exercised 3 times a week for 40 min each time, which was arranged in the morning of Mondays, Wednesdays, and Fridays respectively, in which they prepared for activities and relaxed for about 15 min. The objects of the same gender had the same content of preparation and relaxation activities. The exercise intensity was controlled at medium level, and the monitoring method was to randomly select individual subjects every 10 min to measure their immediate heart rate for 10 s during the exercise process. The heart rate of the objects during the exercise process was controlled at 50–80% of the maximum heart rate of the objects, about 135–150 min. The control group was not required to participate in sports, only after the end of the experiment, the level of inferiority, self-esteem, and general self-efficacy of their basic indicators were measured with observing the change of each variable during the experiment time, to investigate whether there is a significant influence of factors other than experimental conditions.

#### Experiment time and test

The experiment ran for 12 weeks from August 10 to November 10, 2021. Before the experiment, the objects were tested for inferiority, self-esteem, and general self-efficacy. After 6 and 12 weeks, the objects’ sense of inferiority, self-esteem, and general self-efficacy were postmeasured, respectively. Group test method was used to instruct the subjects to fill in the questionnaire.

### Testing tools

The testing tools included sense of inferiority scale, self-esteem scale, and general self-efficacy scale:

#### Sense of inferiority scale

The scale consists of self-respect, social confidence, academic ability, physical appearance, and physical fitness. The sum of the five dimensions indicates total sense of inferiority. Likert5 scores range from 1 for “not at all” to 5 for “always.” The higher the test score is, the lower the self-score is, the weaker the sense of inferiority is Ye et al. (2020). A self-reported questionnaire designed to measure inferiority of college students over a period of time. In order to facilitate students to make self-assessment, the original 7 rating scales were changed to 5 in this study. In this study, the reliability and validity of the scale are good, and the internal consistency coefficient of each subscale is 0.80. The retest coefficient is between 0.71 and 0.79, and the retest coefficient of the total amount table is 0.79.

The reliability indexes of college students’ sense of inferiority scale are shown in Table 3.

**Table 3:** Reliability indexes of college students’ sense of inferiority scale.

Reliability indexes	Physical appearance	Physical fitness	Social confidence	Academic ability	Self-respect	Total table
Reliability coefficient α	0.75	0.78	0.74	0.79	0.75	0.80
Retest reliability	0.75	0.77	0.72	0.78	0.76	0.79

#### Self-esteem scale

This scale consists of 10 questions, and the 4-level score is very inconsistent, inconsistent, consistent, very consistent. Scale scores range from 10 to 40, with high scores representing high self-esteem. The version of He and Aishu (2018) was used in this study, and its reliability coefficient was 0.75 in this study.

#### General self-efficacy scale

The general self-efficacy scale developed by Schwarzer was used with 10 questions (Ren et al., 2021). The reliability and validity of the scale have been confirmed in a large number of studies. Many studies using this scale have consistency coefficients between 0.75 and 0.91. The scale is not only concise and reliable, but also has good aggregative and discriminative validity. The scoring method is lickett’s 5-level scoring method. The higher the score is, the higher the general self-efficacy is. In this study, its reliability coefficient is 0.79.

### Reliability check

The reliability of the scale in this study is ideal as shown in Table 4. The average A coefficient of each subscale is between 0.71 and 0.78, and the reliability coefficient of the scale is above 0.70, indicating that the reliability of the scale is acceptable (Xiaoyan et al., 2019). The reliability test results of each submeter are shown in Table 4.

**Table 4:** Reliability test results of each submeter.

Variable	Before experiment	During experiment	After experiment	Average
Physical appearance	0.70	0.71	0.72	0.71
Physical fitness	0.70	0.72	0.75	0.72
Social confidence	0.75	0.77	0.79	0.77
Academic ability	0.77	0.78	0.78	0.78
Self-respect	0.75	0.77	0.77	0.76
General self-efficacy	0.75	0.79	0.78	0.77
Self-esteem	0.75	0.76	0.75	0.75
Sense of inferiority	0.79	0.75	0.76	0.77

## Research results

### Test result

The average and standard deviation of inferiority and its dimensions in control group, competition group and entertainment group were compared before, during and after the experiment. The average and standard deviation of each variable in each test are shown in Table 5.

**Table 5:** Average and standard deviation of each variable in each test.

Variable	Measuring phase	Competition group		Entertainment group		Control group	
		Average	Standard deviation	Average	Standard deviation	Average	Standard deviation
Physical appearance	Before	14.10	3.04	14.58	3.32	14.03	2.82
	During	13.77	2.39	13.92	2.16	13.98	2.85
	After	13.62	2.14	13.81	2.18	13.94	2.02
Physical fitness	Before	14.04	3.05	14.50	3.17	14.02	3.59
	During	13.35	2.17	13.49	2.44	14.25	3.06
	After	11.98	2.45	12.00	2.14	14.08	3.59
Social confidence	Before	30.35	3.52	29.88	3.84	30.48	3.17
	During	27.50	2.82	27.10	2.93	30.05	2.17
	After	25.63	2.08	26.13	2.17	30.16	2.44
Academic ability	Before	20.45	3.01	20.65	3.28	21.00	3.46
	During	19.20	3.17	19.30	3.48	20.76	3.61
	After	18.04	2.56	18.45	2.88	20.24	3.18
Self-respect	Before	26.50	1.08	26.40	1.28	26.63	1.07
	During	25.54	1.15	25.34	1.75	26.20	1.54
	After	25.03	1.63	25.18	1.64	26.31	1.08
Sense of inferiority	Before	105.44	13.7	106.01	14.89	106.16	14.11
	During	99.36	11.7	99.15	12.76	105.24	13.23
	After	94.3	10.86	95.57	11.01	104.73	12.31

As can be seen from the data in Table 5, compared with the control group, the entertainment group had varying degrees of change in all dimensions. Generally speaking, the average score of sense of inferiority and its 5 dimensions showed a downward trend with the experimental time. The average score of the competition group was lower than that of the entertainment group, and the entertainment group was lower than that of the control group. In general, the average score of inferiority in the competition group was lower than that in the entertainment group, while that in the entertainment group was lower than that in the control group.

### Multivariate equation analysis

The inter-group comparison results of multivariate variance analysis on college students’ inferiority feeling under different sports scenarios are shown in Table 6.

**Table 6:** Inter-group comparison results of multivariate variance analysis on college students’ inferiority feeling under different sports scenarios.

Dependent variable	Group name		*M*	*D*	Sig.
Physical appearance	Competition group	Entertainment group	−1.69	0.725	0.118
	Competition group	Control group	−0.86	0.715	0.224
	Entertainment group	Control group	0.88	0.762	0.237
Physical fitness	Competition group	Entertainment group	0.31	0.820	0.563
	Competition group	Control group	−1.38	0.762	0.039
	Entertainment group	Control group	−1.83	0.820	0.016
Social confidence	Competition group	Entertainment group	1.32	1.263	0.244
	Competition group	Control group	−0.14	1.239	0.018
	Entertainment group	Control group	−1.62	1.183	0.048
Academic ability	Competition group	Entertainment group	0.78	0.936	0.514
	Competition group	Control group	−0.32	0.925	0.045
	Entertainment group	Control group	−1.20	0.832	0.074
Self-respect	Competition group	Entertainment group	−0.76	0.840	0.323
	Competition group	Control group	−0.31	0.825	0.067
	Entertainment group	Control group	0.21	0.843	0.069
Sense of inferiority	Competition group	Entertainment group	−0.04	4.584	1.762
	Competition group	Control group	−3.01	4.466	0.393
	Entertainment group	Control group	−3.56	4.440	0.444

This study further investigated the influence of sports on college students’ sense of inferiority by means of variance analysis. After the experimental college students’ sense of inferiority and its dimensions as the dependent variable, scenarios, such as the independent variable to single factor multivariate analysis of variance, in front of the anova for each variable to the f test, results show that sense of inferiority, self-respect, social confidence, academic ability, physical appearance, and physical fitness are in the current degree of freedom, the *p* < 0.05. In the competition group and the entertainment group, the mean differences in the other dependent variables were statistically significant (*p* < 0.05), except for the mean differences in respect and appearance between the competition group and the control group (*p* < 0.05). In terms of the difference between groups, the scores of the experimental group on various dependent variables decreased after the experiment, indicating that sports can indeed reduce the sense of inferiority of college students, especially in competitive, and recreational situations.

### Before and after comparative analysis

This study intends to use the experimental method to investigate the variation trend of each predictive variable and sense of inferiority in the whole experimental process, so as to further verify this model. *T*-test was conducted on college students’ sense of inferiority and its predictive indexes in different groups at different sports experiments, and the results are shown in Table 7.

**Table 7:** Mean and standard deviation of the test and its relation to the pre-experiment *T*-test.

Variable	Measuring phase	Competition group			Entertainment group			Control group		
		Average	Standard deviation	*T*	Average	Standard deviation	*T*	Average	Standard deviation	*T*
General self-efficacy	Before	23.79	5.45	–	23.64	4.62	-	23.63	5.07	-
	During	28.05	6.26	−1.04	27.55	5.39	−1.84	23.71	5.04	−0.19
	After	32.54	7.28	−2.63	33.62	5.08	−2.64	23.99	5.07	−0.24
Self-esteem	Before	23.30	2.28	-	23.67	1.65	-	23.13	1.85	-
	During	29.90	2.45	−0.29	26.30	2.14	−1.08	23.35	2.58	−0.06
	After	29.65	5.24	−2.59	28.02	3.69	−2.04	23.73	2.62	−0.86
Sense of inferiority	Before	106.14	14.26	-	106.52	15.23	-	106.74	15.09	-
	During	98.52	11.78	13.65	99.65	11.74	10.63	105.82	14.69	1.18
	After	93.04	10.63	21.09	94.05	11.18	20.63	105.33	14.65	1.75

In the 6th week of the experiment, the score of inferiority in tennis was statistically significant (*p* < 0.05), and the score of general self-efficacy and self-esteem was improved but not statistically significant. After the experiment, the scores of sense of inferiority, self-esteem and general self-efficacy were statistically significant with the *T*-test results before the experiment. The scores of inferiority continued to decrease, while the scores of self-esteem and general self-efficacy continued to increase. Throughout the experiment, the scores of sense of inferiority decreased with the increase of experiment time, while the scores of self-esteem and general self-efficacy increased with the increase of experiment time. The variation trend of these variables over time is consistent with the prediction model. The scores of sense of inferiority, self-esteem, and general self-efficacy in the entertainment group were similar to those in the competitive situation.

### Discussion on the results

Before grouping, this study measured the sense of inferiority of the objects, its predictive indicators and physical activity, and then matched the groups. Through the 12-week experiment, the results confirmed the research hypothesis. At the same time, the multivariate nonlinear regression model of the relationship between sports activities and sense of inferiority was further verified by investigating the variation trend of relevant variables under the experimental control. In general, sports activities play a positive role in reducing college students’ sense of inferiority. This is similar to the results of de Souza et al. (2021) that more exercise is good for students’ mental health.

1. The inferiority of college students is more prominent, targeted sports should have a significant impact on their mental health. The sports scene created by this research is just in line with this requirement. The scene creation has a certain connection with the happy learning and successful experiential learning advocated by the current higher education reform, and the experimental control is easier to implement. The experimental results also provide a certain support for the higher education PE and health curriculum reform.
2. In this study, competitive sports scenarios and recreational sports scenarios were created. Both sports scenarios had positive effects on college students’ inferiority feeling, and reached statistical significance. Through this kind of competitive sports situation, one can detect and develop the internal drive of one’s own ability, and the acquisition of this sense of ability and self-determination can bring a positive effect. In the context of recreational sports, sports participants can not only enhance their physical fitness and improve their health, but also relax their highly nervous nerves, stabilize their mentality, and gain self-confidence, pride, and a sense of achievement in sports. Muñoz-Bullón et al. (2017) pointed out in his research that sports promote academic performance. We believe that sports can lead to higher academic achievement by reducing students’ sense of inferiority.
3. In terms of the benefits of sports’ influence on sense of inferiority, the medium intensity sports selected in this study showed higher benefits. Regardless of the competition group or the entertainment group, after 12 weeks of sports, the level of inferiority feeling of the objects was reduced to varying degrees, and it was statistically significant. This further confirms the psychological benefits of sports, indicating that appropriate sports activities have positive significance to promote the mental health of college students.
4. In different sports scenarios, similar results were obtained despite different degrees. With the increase of sports time, the changes of each variable were basically consistent with the changes in the prediction model, thus proving the suitability of the model. For the mediators, because they are non-linear mediators, the validation of them cannot be discussed by simple correlation. What matters in the model is that self-esteem and general self-efficacy are enhanced by sports activities and reduced by sense of inferiority. It shows that under the condition of the control of this experiment, the results of the influence of sports activities on variables are basically consistent with the expected assumption.

## Conclusion

Situational factors should be considered in the formulation of exercise prescriptions for the effects of physical exercise on mental health. This study investigates the influence of sports on college students’ inferiority through multiple linear regression model. After F-test, analysis of variance was performed for each variable. The results showed that inferiority, self-esteem, social confidence, academic ability, appearance, and physical quality were all under the current degree of freedom. 0.05. The experimental results show that physical exercise is an effective way to reduce the sense of inferiority. In addition, we also study the similarities and differences of the influence of competitive sports and leisure sports on students’ inferiority. In terms of the influence of the sports situation created by this study on college students’ sense of inferiority, the competitive sports situation is better than the recreational sports situation. Different psychological variables have different requirements for the duration of exercise, and different duration of exercise has different effects on college students’ sense of inferiority. Generally speaking, the longer the duration of exercise, the more significant the effect of reducing sense of inferiority. This suggests that the duration of exercise program should be determined according to different psychological variables when making exercise prescription about the influence of physical exercise on mental health. Through the theoretical and empirical research on college students’ sense of inferiority, this study has obtained satisfactory results, obtained some meaningful conclusions. Firstly, this study enriches the research on college students’ inferiority complex. The psychological health of college students has been concerned by many scholars. This paper expands the research field of college students’ inferiority by studying the influence of sports on college students’ inferiority. Secondly, we took self-esteem and general self-efficacy as research variables to explore the mediating effect of these two sports on college students’ feelings of inferiority. Therefore, we expanded the research field of sports, self-esteem, general self-efficacy and inferiority. Finally, we created different sports situations and discussed the influence of competitive sports situations and leisure sports situations on the reduction of college students’ inferiority complex. Therefore, this study is helpful for university teachers to formulate appropriate strategies. This strategy is effective in reducing college students’ inferiority complex. And also found some problems worth exploring. Due to the limitation of manpower, material resources and time, there are still some problems and deficiencies in some aspects, which need to be further and further studied in the future. In this study, due to the limitations of various conditions, it is difficult to conduct a wider range of sampling in the subject sampling, so it is necessary to conduct in-depth and comprehensive research on this issue in the future. Second, in this study, the research samples are from Chinese students of Northeast Normal University. However, due to the cultural and teaching differences at home and abroad, it is worth discussing the similarities and differences between Chinese college students and American/European college students in the future.

## Data availability statement

The original contributions presented in the study are included in the article/supplementary material, further inquiries can be directed to the corresponding author.

## Author contributions

The author confirms being the sole contributor of this work and has approved it for publication.

## Conflict of interest

The author declares that the research was conducted in the absence of any commercial or financial relationships that could be construed as a potential conflict of interest.

## Publisher’s note

All claims expressed in this article are solely those of the authors and do not necessarily represent those of their affiliated organizations, or those of the publisher, the editors and the reviewers. Any product that may be evaluated in this article, or claim that may be made by its manufacturer, is not guaranteed or endorsed by the publisher.
